# Values in Action: Unveiling the Impact of Self-Transcendence and Self-Enhancement on Domestic Consumption Choices

**DOI:** 10.3390/bs14030203

**Published:** 2024-03-04

**Authors:** Zerui Zhao, Lu Huang

**Affiliations:** Business School, Sichuan University, Chengdu 610065, China; zhaozeruivip@126.com

**Keywords:** self-transcendence, self-enhancement, domestic-product consumption, personal values, consumer behavior, norm-activation model

## Abstract

Against the backdrop of a global emphasis on supporting local businesses and fostering domestic consumption, this study aims to shed light on the influence of personal values on the intentions behind domestic-product consumption. Drawing from the Schwartz value theory, we explore how values of self-transcendence, which embody benevolence and universalism, versus self-enhancement, characterized by a focus on power and achievement, influence consumer behavior. Utilizing data from the Chinese Social Survey (CSS2021) and a survey of 316 participants, structural equation modeling and Dematel analysis are employed to reveal causal relationships between values and consumption intentions. We reveal a dichotomous impact of these value orientations. Self-transcendence values are found to positively affect domestic consumption intentions by enhancing awareness of consequence and ascription of responsibility, thereby strengthening personal norms. In contrast, self-enhancement values tend to impede these intentions. By integrating the Norm-Activation Model (NAM), this study comprehensively uncovers the unique mechanism through which values activate personal norms and subsequently encourage the consumption of domestic products. It enriches the body of research related to values and domestic consumption and offers pertinent recommendations for promoting local enterprises’ products.

## 1. Introduction

In 2021, President Joe Biden of the United States signed an executive order aimed at “ensuring a prosperous future for American workers nationwide”. Similar initiatives have been launched in various countries and regions, including Australia, Indonesia, Vietnam, and China [1], reflecting the growing trend of safeguarding domestic businesses and promoting domestic consumption. According to the “2022 China Consumption Trend Report” by Zhimeng Consulting, 42.5% of Chinese consumers increased their purchases of domestic products in 2021. The “2023 McKinsey China Consumer Report” by McKinsey & Co. also indicates that local Chinese enterprises are progressively gaining ground in the domestic consumer market, with a growing preference for domestic products. The primary driver of this trend is the favorable ”price–performance ratio” of domestic products coupled with the desire to ”support local enterprises”. This reflects a shift in the values and attitudes of Chinese consumers toward more rational consumption, heightened environmental awareness, and a preference for ”long-termism”. In this context, the influence of personal values on the purchasing decisions concerning domestic products, as well as the underlying mechanisms, warrant in-depth investigation.

The consumption of domestic products not only exemplifies prosocial and pro-environmental behaviors but also stands as a tangible expression of socially responsible consumption (SRCB). This practice fosters the growth of local industries, stimulates economic expansion, and creates job opportunities. Moreover, purchasing local products reduces transportation distances and significantly cuts carbon emissions.

Extensive academic validation supports the notion that values play a pivotal role in predicting pro-social behavior, with a strong correlation between personal values and pro-social behavior [2,3,4]. Surprisingly, only a few studies explore how personal values, as a form of pro-social behavior, affect the propensity to consume domestic products. Past studies on pro-environmental behavior [5,6], socially responsible consumption behavior [7,8,9], and other dimensions of the role of personal values have been fully validated. Studies have also emphasized the role of personal values in driving consumer behavior [10,11,12,13,14]. However, some studies do not account for the intrinsic mechanisms by which values influence consumer behavior, such as the study of SRCB [7,9]. Furthermore, the existing literature primarily frames the influence of values on the consumption of domestic products within the narrow confines of individualistic and collectivistic values [8], significantly limiting the breadth of the discussion. Hence, this paper introduces the Schwartz value theory, which is more universal and captures the human value system more holistically [15,16], to explore how consumers’ values influence their domestic-product-consumption intention and illustrate its underlying mechanisms.

Normative belief is an important antecedent influencing consumer behavior [17], with numerous studies highlighting the impact of social norms on consumption intentions [18,19,20,21]. However, personal norms are often shown to play a more direct role in shaping consumer choices [22,23,24,25]. This study posits that the effect of consumers’ values on their intentions toward domestic-product consumption is mediated through personal norms. The process of personal norm formation is significantly influenced by individual values [26,27,28,29]. To explore this relationship, this paper employs the Norm-Activation Model (NAM) as the mediating framework. Specifically, we introduce the mediation model of NAM as the mediation mechanism of the study, which, in turn, simultaneously verifies the applicability of NAM’s mediation model in the domestic consumption scenario. Compared with previous studies, this paper not only focuses on the role of personal norms but also explores the dynamic process of personal norm formation. This provides a novel lens through which to examine the intrinsic mechanisms by which values affect consumer behavior, offering insights beyond those of previous studies that have largely focused on the role of personal norms without exploring their formation process in depth.

The main contents include: (1) Reviewing existing studies on values, norm-activation theory, and domestic-product-consumption intentions, and formulating research hypotheses. (2) Conducting exploratory research using data from the 2021 Chinese Social Survey to assess the impact of personal values on domestic-product-consumption intentions. (3) Applying structural-equation analysis to the survey data to explore how personal values affect domestic-product-consumption intentions through the norm-activation model, drawing research conclusions, and explaining the significance of the research.

The theoretical contributions of this study are threefold: (1) This study verifies the contrasting effects of “self-enhancement” and “self-transcendence” values on the consumption intention of domestic products, thus broadening the research on the antecedents of the influence of domestic consumption. (2) The norm-activation theory was introduced to reveal the internal psychological mechanism, and the chain-mediating role of the awareness of consequence, ascription of responsibility, and personal norms was verified. The unique mediating mechanism of the dynamic process of norm activation is explored. (3) The applicability of the NAM mediator model in domestic consumption scenarios is verified through structural equations and the Dematel causality test, enriching related studies that have utilized the norm activation theory.

## 2. Literature Review and Hypothesis Development

### 2.1. Values and Domestic-Product Consumption Intention

#### 2.1.1. Domestic-Product Consumption Intention

Domestic-product-consumption intention refers to a consumer’s preference for purchasing domestic products when making buying decisions. This intention reflects a commitment to supporting and developing the domestic industry, often viewed as an expression of patriotism and, thus, a form of socially responsible consumer behavior.

In the realm of factors influencing domestic-product-consumption intentions, consumer ethnocentrism is widely acknowledged as a significant factor. This concept implies a normative belief that buying foreign products is inappropriate and that purchasing domestic items supports local firms [17,30]. While many studies have established a causal link between consumer ethnocentrism and domestic-product consumption [17,31,32,33], there is some inconsistency in these findings. For instance, certain studies indicate that the relationship between ethnocentrism and domestic-product consumption may only be partially correlated [34,35].

Incorporating the effects of other elements can help reconcile these varying perspectives. Klein et al., in their study within the Chinese context, found that hostility toward foreign products extends beyond the scope of consumer ethnocentrism, adversely affecting the purchase of such products [33,36,37]. Similarly, He and Wang’s research revealed that cultural identity strengthens the preference for domestic brands and diminishes the appeal of imported ones, suggesting that consumer ethnocentrism mainly manifests in the boycotting of foreign products [38]. Zeugner-Roth et al. proposed a comprehensive model grounded in the social identity theory, identifying consumer nationalism, national identity, and consumer cosmopolitanism as key predictors of domestic-product-consumption decisions [39]. Moreover, Gineikiene et al.’s work highlights the role of psychological ownership in determining preferences for domestic versus foreign products, suggesting that domestic psychological ownership might better explain these decisions than consumer ethnocentrism [33,40].

Additionally, the influence of social norms has been a focus of many studies. Research by Granzin and Painter in Portugal and the USA found that social norms significantly predict domestic purchasing behavior [18]. Similar findings were observed in studies conducted in China and South Africa [19,21]. These studies concluded that social norms impact domestic-product-consumption intentions through four pathways: direct, motivational, cognitive, and motivational–cognitive [21]. However, existing research often examines the effect of norms on consumption intentions in isolation. This paper posits that differing values influence the internalization of social norms, and thus, considering the norm formation process as a mediating mechanism in domestic-product-consumption intention can be insightful. Evaluating the effect of values on norms could offer a robust prediction of domestic-product-consumption intentions.

Other research has explored the influence of factors like perceived financial status [41], emotions, demographic characteristics [42], social media activities [43] and brand innovativeness [44]. Collectively, these studies encompass a range of factors from consumer psychology, social norms, and culture to the types and attributes of products. However, many of these studies present varying degrees of controversy. This paper suggests that exploring the impact of individual consumer values can enrich the existing body of research and offer a fresh perspective to reconcile the debates among current studies.

#### 2.1.2. Values and Domestic-Product Consumption Intention

Values are broad, aspirational goals that inspire actions and influence perception, cognition, and behavior as guiding principles in life. Currently, the Schwartz value theory is the most mature and widely accepted theory in the academy, which posits that values are persistent beliefs related to desirable end-states or behaviors [45,46] and are desirable transcendent situational goals that act as guiding principles in a person’s life or other social existence [46,47].

A large number of studies have proved that there is a high correlation between personal values and their corresponding behaviors [46,48]. For example, Schwartz pointed out the influence of values on pro-social behavior. According to the categorization in Schwartz’s value model, self-transcendence values relate to people’s pro-social behaviors in different cultures and contexts [46,49]. For example, scholars have investigated its effects on everyday acts of kindness in the Netherlands, Israel, Italy, Poland, Russia, the United Kingdom, and the United States and have found that people with self-transcendent values are more likely to be charitable, share, and help others [10,50,51]. There is also evidence of a causal relationship between values and pro-social behavior, with experimental participants with more kind and benevolent values showing more pro-social behavior [2,3,52]. Similar findings have been validated in experiments related to pro-environmental behavior [53,54].

Based on Schwartz’s model of values, “self-transcendence” characterizes the values of equal acceptance of others and concern for the interests of others as being more concerned with social outcomes as opposed to “self-enhancement”, which is more concerned with individual outcomes and emphasizes the pursuit of personal success and control over others [3,46]. When an individual’s motivation to act puts more emphasis on the pursuit of individual success and the achievement of self-enhancement, his or her motivation to help others, to serve the community, and to achieve self-transcendence will be hindered.

Research has captured the dichotomy between self-transcendence and self-enhancement values in pro-social behavior [55,56,57]. For example, Schultz and Zelezny’s experiment found positive correlations between self-transcendence values and behaviors such as recycling, water and energy conservation, and public transit use, with self-enhancement values showing the opposite. Studies on antisocial behavioral contexts have also found opposing correlations [58,59,60].

Past research has emphasized the role of personal values in driving consumer behavior [10,11,12,15], but there has been insufficient research on how values influence consumers’ consumption intention of domestic products. Some scholars have discussed the effects of collectivist versus individualist values on the purchase of local food, suggesting that collectivist values increase consumer preference for local products [8,61], but the research scenario is limited [62]. In this paper, we place the research scenario in a more generalized national-product-consumption scenario, which can well complement the existing research findings. Also, Schwartz’s value theory represents a more universal representation of human values across cultures, with a broader scope of values than individualism and collectivism, reflecting a more fundamental human value system [47,63,64]. Based on the review and derivation of the ideas in the literature mentioned above, the consumption of national products is essentially a pro-social behavior. Self-transcendence values are believed to promote pro-social behavior, while self-enhancement values are believed to do the opposite. This paper argues that self-transcendence values positively affect consumers’ domestic-product-consumption intentions. In contrast, self-enhancement values negatively affect consumers’ domestic-product-consumption intentions, and establish the research hypothesis accordingly:

**H1a.**  *Self-enhancement values negatively predict consumers’ domestic-product-consumption intentions.*

**H1b.**  *Self-transcendence values positively predict consumers’ domestic-product-consumption intentions.*

### 2.2. Values, Norm Activation, and Domestic-Product Consumption Intention

#### 2.2.1. Norm-Activation Theory

Schwartz proposed that personal values and norms are the sources of motivation for helping behavior, and their activation promotes the formation of an individual’s internal sense of moral obligation, i.e., personal norms, which led to the Norm-Activation Model (NAM) [22].

In this context, Schwartz asserts that personal norms directly influence the occurrence of pro-social behavior. According to NAM, the activation of personal norms begins with social norms. While past research has extensively explored the effect of social norms on domestic-product-consumption intentions [18,19], it is crucial to distinguish personal norms from social norms. Schwartz highlights that social norms can only influence individual behavior when internalized into personal norms at the individual level [22]. Hence, in our study, we introduced the NAM to assert that personal values shape the formation and activation of personal norms, which, in turn, influence consumers’ domestic-product-consumption intentions.

This approach to understanding personal norms and values is supported by a wealth of research across various contexts. The Norm-Activation Model has been validated in numerous studies examining pro-social behaviors and intentions, such as responsible consumption [65], volunteering [66], and various pro-environmental behaviors [60,67]. Furthermore, the integration of additional variables into the NAM in different studies has led to the development of expanded models, solidifying the NAM’s role as a versatile and robust framework for studying pro-social behaviors [68,69]. Overall, the Norm-Activation Model has become a reliable tool for studying pro-social, altruistic, and responsible consumption behaviors and has been repeatedly validated and widely accepted by the academic community. This paper also establishes a research model based on the NAM.

The Norm-Activation Model proposes three variables to predict pro-social behavior [22]. The first is Personal Norms (PN), referred to as “moral obligations to perform or refrain from specific actions” [70], and is the central variable of the NAM. The second is Awareness of Consequence (AC), which refers to an individual’s recognition of the negative impact on others or the consequences of failing to take socially beneficial actions [70]. The third, Ascription of Responsibility (AR), involves acknowledging personal responsibility for the adverse outcomes resulting from the absence of pro-social behavior [70].

De Groot proved the reliability of the mediation model of the NAM through multiple tests in different contexts, and their study showed that a person must be aware of the consequences of behavior before being held responsible for it, meaning that awareness of consequence leads to the ascription of responsibility. Personal norms are activated by ascriptions of responsibility, and then personal norms induce behaviors [70]. The mediation model of the NAM is now widely recognized in the academic community, and the research hypotheses established in this paper are based on this model.

#### 2.2.2. The Mediating Role of Personal Norms

Personal norms represent an individual’s internalization of social norms, manifested as a sense of moral obligation [22]. As the central variable of the Norm-Activation Model (NAM), personal norms play a crucial role in determining whether an individual engages in pro-social behaviors. The presence of a strong moral obligation often leads individuals to align their actions with their deeply held values, thereby fostering appropriate pro-social behaviors.

Self-transcendence values are more concerned with the welfare and interests of others (expressed as universalism and benevolence values) and more concerned with social outcomes as opposed to self-enhancement values (expressed as power and achievement values), which are more concerned with personal outcomes such as self-interests, relative success, and so on [46]. Extensive research has established that self-transcendence values are positively correlated with pro-social and altruistic behaviors [10,51,71], whereas self-enhancement values typically show a negative association with these behaviors [2,52]. Based on the above, we propose the following hypotheses:

**H2a.**  *Self-enhancement values negatively predict personal norms.*

**H2b.**  *Self-transcendence values positively predict personal norms.*

**H3a.**  *Personal norms mediate the negative influence of self-enhancement values on domestic-product-consumption intention.*

**H3b.**  *Personal norms mediate the positive influence of self-transcendent values on domestic-product-consumption intention.*

#### 2.2.3. The Mediating Role of Awareness of Consequence

Awareness of consequence is the awareness that an individual’s failure to perform an altruistic behavior will negatively affect others or other things [72,73]. When a person is aware of the consequences of their actions, they feel a strong obligation to act in a certain way [74]. Numerous prior studies have demonstrated that a person’s awareness of consequence is positively correlated with the level of their personal norms in pro-social behaviors [69]. We propose that the stronger an individual’s perception of consequences, the more intense their sense of moral responsibility becomes, thus increasing the likelihood of activating personal norms to engage in appropriate pro-social behaviors.

Suppose that personal values are more inclined to engage in pro-social behaviors. In that case, individuals are more likely to attribute negative consequences to themselves when they become aware of the consequences of their non-pro-social behaviors [70,75,76]. For instance, when an individual’s level of “self-transcendence” values is high, characterized by benevolence and universalism, they are more apt to perceive and be affected by the consequences of behaviors detrimental to the collective interest. This heightened perception facilitates the stimulation of their sense of moral responsibility, subsequently increasing the probability of purchasing domestic products. Conversely, a higher level of “self-enhancement” values, which focus more on individual success and personal gain, tends to diminish this perception and the consequent sense of moral responsibility. Based on these insights and the mediation model of NAM, we propose the following hypothesis:

**H4a.**  *Awareness of consequence and personal norms play a chain-mediating role in the negative influence of self-enhancement values on domestic-product-consumption intention.*

**H4b.**  *Awareness of consequence and personal norms play a chain-mediating role in the positive influence of self-transcendence values on domestic-product-consumption intention.*

#### 2.2.4. The Mediating Role of Ascription of Responsibility

Ascription of responsibility refers to individuals’ feelings of responsibility when their actions lead to negative consequences [69,70,72]. As an activator of personal norms, ascription of responsibility plays a critical role in generating moral obligations [70]. When individuals become aware of these negative consequences and form ascriptions of responsibility, they are more likely to develop a sense of moral obligation to engage in pro-social behaviors [70,75,76]. Consequently, this awareness increases their willingness to participate in pro-social behaviors [69].

In the context of supporting domestic products, individual behavior that contradicts this principle can result in negative outcomes. Such behavior might evoke feelings of guilt in the individual responsible for it. When he or she acknowledges the need to take responsibility for these negative consequences, there is an increased likelihood of adopting behaviors that align with personal norms [70,77]. Drawing from the mediation model of NAM, we propose that an individual’s ascription of responsibility plays a pivotal role in aligning their behavior with personal norms, particularly in the context of domestic-product consumption. Therefore, we suggest the following hypothesis:

**H5a.**  *Awareness of consequence, ascription of responsibility, and personal norms play a chain-mediating role in the negative influence of self-enhancement values on consumers’ domestic-product-consumption intention.*

**H5b.**  *Awareness of consequence, ascription of responsibility, and personal norms play a chain-mediating role in the positive influence of self-transcendence values on consumers’ domestic-product-consumption intention.*

**Figure 1:**
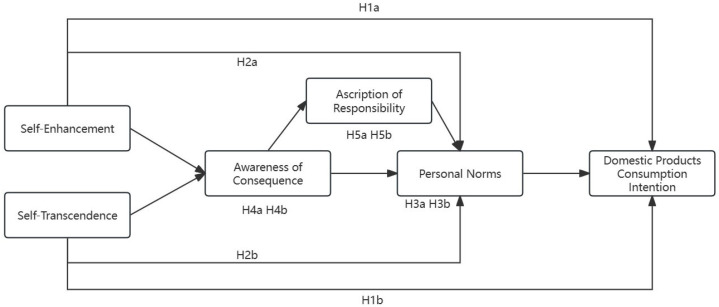
Research model.

Based on the above theories and our research hypotheses, we constructed a model of the influence of values on consumers’ domestic-product-consumption intention, as shown in Figure 1.

## 3. Study 1

### 3.1. Materials and Methods

Study 1 is an exploratory study designed to preliminarily validate the main effect of this research, namely, the impact of values on consumers’ domestic-product-consumption intentions. This study utilized data from the 2021 Chinese Social Survey (CSS2021).

The China Social Situation Survey (CSS), initiated by the Institute of Sociology at the Chinese Academy of Social Sciences (CASS) in 2005, is a nationally representative household survey conducted biennially. It aims to gather data on and insights into the social transformations occurring in China during its transition period. By conducting long-term longitudinal studies on the public’s labor and employment, family life, social interactions, and attitudes, the CSS seeks to furnish valuable and scientifically grounded information for social-science research and policy-making.

The 2021 iteration, marking the eighth phase of the survey, completed household interviews across 592 villages in 30 provinces, municipalities, and autonomous regions throughout China, amassing 10,136 valid questionnaires. The data was officially released to the public on 31 December 2022 (see Supplementary Material for data access).

The dimensions of self-enhancement values in Schwartz’s value theory including ”power” and ”achievement” [46], were measured in this study. We referred to the relevant question in the Schwartz Value Scale that pertains to self-enhancement values, deducing that a preference for power and wealth signifies these values. In CSS2021, individuals who selected the third option in question D7b, “What do you think best describes a person’s values?—Being more powerful or wealthy than others” were identified as having self-enhancing values. Consistent with Schwartz’s value theory, which positions self-enhancement and self-transcendence values at opposite ends [3], individuals with high self-enhancement values are presumed to have lower self-transcendence values, and vice versa. Therefore, participants who did not choose the third option in question D7b are considered to possess higher self-transcendence values. Additionally, the question item D9B-5, “I favor national brands” was utilized to gauge domestic-product-consumption intentions in this study. The variable measurement items employed in this study are detailed in Table 1, based on the data availability.

### 3.2. Results

The study utilized SPSS 25.0 to test the hypotheses by conducting independent sample *t*-tests on the data. Among the 10,136 samples of CSS2021, 8456 samples did not answer both the D7a and D9b-5 questions, or the answer was “I can’t say”. Based on the availability of data, the remaining 1680 samples that answered the above questions were finally selected, and the results are shown in Table 2.

The mean scores of domestic-product-consumption intention of the samples with self-enhancement and self-transcendence were 2.476 ± 0.750 and 2.973 ± 0.746, respectively. The *t*-test results (*t* = −3.1033, *p* < 0.01) show that the difference in domestic-product-consumption intention between individuals with self-enhancement and self-transcendence values is statistically significant, and that individuals with self-enhancement values have a lower domestic-product-consumption intention. Individuals with self-transcendence values have a higher domestic-product-consumption intention, and the test results support H1a and H1b.

The exploratory study utilizing CSS2021 data revealed that self-enhancement values tend to negatively influence domestic-product-consumption intentions, whereas self-transcendence values have a positive impact. However, it is important to note that while the CSS2021 questionnaire items provide some insights into personal values, they do not constitute an in-depth examination of these values in isolation. The survey’s measurement items may not fully and accurately capture the nuanced meanings of the value dimensions as conceptualized in Schwartz’s value theory. Additionally, the exploratory study’s methodology limited its ability to delve into the underlying mechanisms by which values affect domestic-product-consumption intentions. Consequently, Study 2 was conceived to provide a more comprehensive analysis.

## 4. Study 2

### 4.1. Sample and Data Collection

The data for Study 2 were derived from a questionnaire survey conducted among consumers in China. The survey was administered via the Credemo platform, which enabled participants to complete the questionnaire online. Access to our survey was facilitated through the Data Mart feature on Credemo. At the outset of the questionnaire, we outlined the purpose of the data collection and its scope of application, ensuring that participation was entirely voluntary. Out of 349 distributed questionnaires, 33 responses were discarded due to rapid completion times, evident errors, or multiple submissions from the same individual, yielding 316 valid questionnaires (validity rate: 88.22%). The sample size of our study exceeded ten times the number of questionnaire items and was considered adequate [78,79,80,81]. The essential characteristics of the sample were analyzed, with the demographic information about the respondents presented in Table 3.

### 4.2. Instruments

The questionnaire consisted of three sections. There were 13 items measuring values, all of which were selected from the “self-transcendence” and “self-reinforcement” values in the Portrait Values Questionnaire (PVQ) [49], which is a commonly used scale in Schwartz’s value theory for the measurement of personal values. This section of the questionnaire was based on a six-point Likert scale, ranging from 1 (not like me at all) to 6 (very much like me).

A total of 11 questions were asked about the mediator variables, which were set up based on previous studies in the context of the Norm-Activation Model. The questionnaire used a seven-point Likert scale from 1–7, with “1” indicating “completely agree” and “7” showing “completely disagree”.

There are three items for measuring domestic-product-consumption intention, all of which were selected from the dimension of “support for domestic products” in the Consumer Willingness to Consume Domestic Products Scale revised by Yan Jun and She Qiuling [82], which has been widely used in the measurement of Chinese consumers’ willingness to consume domestic products. This section of the questionnaire utilizes a seven-point Likert scale from 1–7, with “1” indicating “completely consistent” and “7” showing “completely inconsistent”.

The specific measurement items, the means, standard deviations, factor loadings, and literature sources for each are shown in Table 4.

### 4.3. Results

#### 4.3.1. Reliability Test

The data were first analyzed for the internal consistency of each dimension through SPSS 25.0 using Cronbach’s-coefficient reliability test. The results of the reliability analysis are shown in Table 5. The reliability coefficients for the scales overall and for each dimension were in the range of 0.8–1, thus indicating that the scales used in this study all had good internal consistency.

To test the validity of the measurements, this study conducted a validated factor analysis (CFA). Based on the CFA model fit test results in Table 6, it can be seen that χ^2^/df (chi-square degrees of freedom) = 2.712, RMSEA = 0.066, CFI = 0.922, TLI = 0.914, and IFI = 0.952, which show a good model fit.

The study conducted an analysis of the convergent validity and discriminant validity of the CFA model. A total of six factors were extracted, and validated factor analysis was conducted for the 27 analyzed items. According to Table 7, it can be seen that the AVE values corresponding to the six factors are greater than 0.5, and the CR values are higher than 0.7, indicating that the data of this analysis have good convergent validity. Table 8 shows the HTMT values between the factors, and according to the test results, the overall discriminant validity of the model was found to be good.

#### 4.3.2. Descriptive Statistics

This study used descriptive statistics to examine the relationships between the variables. As shown in Table 9, self-enhancement was negatively correlated with the other variables, and all other variables reflected significant positive correlations with each other. Regarding personal values, the self-enhancement (M = 3.009, SD = 1.252) dimension scored higher relative to the self-transcendence (M = 2.389, SD = 1.027) dimension. Slightly higher scores were found for ascription of responsibility (M = 3.525; SD = 1.587) and personal norms (M = 3.610, SD = 1.786), and marginally lower scores were found for consumption behavior (M = 3.495, SD = 1.743) and awareness of consequence (M = 3.435, SD = 1.457).

#### 4.3.3. Structural Equations

**Figure 2:**
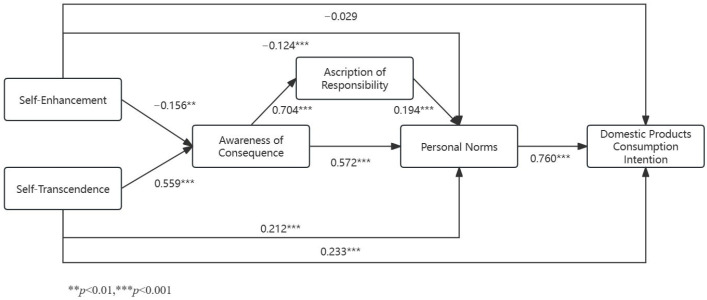
Structural equation model. ** *p* < 0.01, *** *p* < 0.001.

Based on the hypotheses, the study developed a structural equation model and conducted path tests using Analysis of Moment Structure (AMOS) 25.0. The test results are shown in Table 10, and the structural equation model is shown in Figure 2.

The results of the path coefficient test showed that the model was well-fitted (χ2/df = 2.319; RMSEA = 0.065; CFI = 0.944; TLI = 0.938; IFI = 0.944). We found that self-transcendence values had a significant positive effect on awareness of consequence (β = 0.559, *p* < 0.001), personal norms (β = 0.229, *p* < 0.001), and domestic-product-consumption intention (β = 0. 212, *p* < 0.001). Self-enhancement values had a significant negative effect on awareness of consequence (β = −0.156, *p* < 0.01) and a significant negative impact on personal norms (β = −0.124, *p* < 0.001) but not on domestic-product-consumption intention (β = −0.124, *p* = 0.385). Awareness of consequence significantly and positively affected ascription of responsibility (β = 0.704, *p* < 0.001) and also had a significant positive effect on personal norms (β = 0.572, *p* < 0.001). Ascription of responsibility significantly positively affected personal norms (β = 0.194, *p* < 0.001). Personal norms significantly positively influenced consumers’ domestic-product-consumption intention (β = 0.760, *p* < 0.001). According to the above results, H1b, H2a, and H2b are supported.

**Figure 3:**
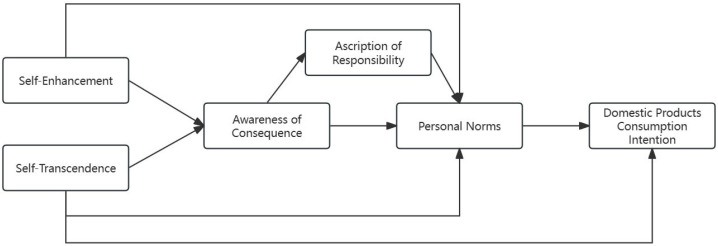
Modified structural-equation model.

According to the path coefficient test results, the modified structural-equation model is shown in Figure 3.

#### 4.3.4. Intermediary Analysis

The study used the bootstrap method in Process to test the mediating effect. Model 6 was chosen to verify the chain-mediating impact of awareness of consequence, ascription of responsibility, and personal norms between the values and domestic-product-consumption intention. Self-enhancement and self-transcendence values were the independent variables, domestic-product-consumption intention was the dependent variable, awareness of consequence was the mediator variable 1 (M1), ascription of responsibility was the mediator variable 2 (M2), and personal norms was the mediator variable 3 (M3); the repeated-measurement sample size was 5000, and confidence intervals were set at 95%.

Self-enhancement values were first analyzed as an independent variable; the results are shown in Table 11. We found that the total effect of self-enhancing values on domestic-product-consumption intentions was significant (Effect = −0.6322, 95% CI = −0.7690 to −0.4955), and the indirect effect through personal norms was significant (Effect = −0.2230, 95% CI = −0.3138 to −0.1419). The series of indirect effects due to awareness of consequence and personal norms were significant (Effect = −0.2106, 95% CI = −0.2890 to −0.1378), and the series of indirect effects due to awareness of consequence, ascription of responsibility, and personal norms were significant (Effect = −0.0727, 95% CI = −0.1162 to −0.0399). Thus, H1a, H3a, H4a, and H5a are supported.

The self-transcendence values were analyzed as an independent variable, and the results are shown in Table 12. We found a significant total effect of self-transcendence values on domestic-product-consumption intentions (Effect = 1.1578, 95% CI = 1.0236 to 1.2921) and a significant indirect effect through personal norms (Effect = 0.3461, 95% CI = 0.0533 to 0.4536). The indirect effects through awareness of consequence and personal norms were significant (Effect = 0.3222, 95% CI = 0.2270 to 0.4164). Through awareness of consequence, ascription of responsibility, personal norms were significant (Effect = 0.0706, 95% CI = 0.0334 to 0.1278). Thus, H1b, H3b, H4b, and H5b were supported.

#### 4.3.5. Dematel Causality Test

**Figure 4:**
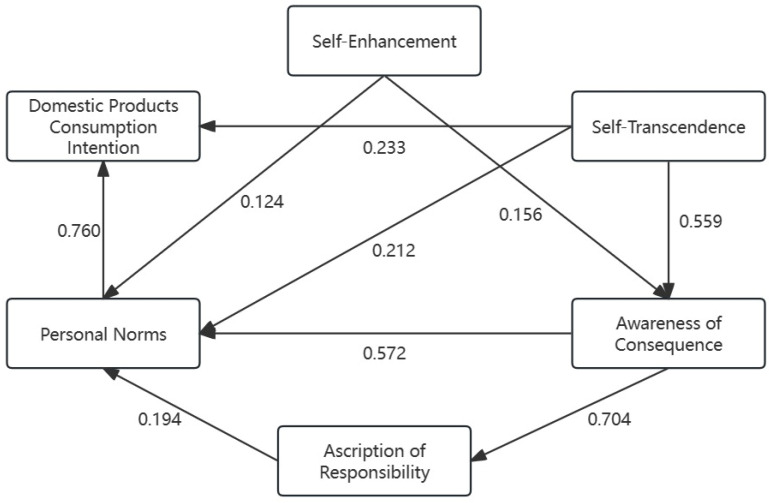
Relationships between variables.

Dematel (Decision-Making Trial and Laboratory) analysis is utilized to establish the logical relationships between model elements by constructing a comprehensive impact matrix. This method enables the determination of both the existence and strength of relationships between elements [86]. In this paper, we employed Dematel analysis via SPSSAU to further test the logical relationships within the model and to validate the model’s structural rationality. Following the exclusion of insignificant variables, the results of the Dematel causality test among the remaining variables are presented in Table 13. Figure 4 illustrates the relationships between variables as identified by the Dematel test, with the numbers on the arrows indicating the magnitude of the effects.

Notably, ascription of responsibility, personal norms, and domestic-product-consumption intention exhibit negative cause degrees, classifying them as influenced factors. Conversely, self-enhancement and self-transcendence values, along with awareness of consequence, demonstrate positive cause degrees, categorizing them as influencing factors. Among these, self-transcendence values, awareness of consequence, and personal norms are particularly central within the system, signifying their critical role and substantial impact on the overall model. These results further corroborate the causal relationships within the model, demonstrating how values influence the activation of personal norms and, ultimately, consumers’ domestic-product consumption intentions.

## 5. Discussion

### 5.1. Theoretical Contributions

The exploration of self-transcendence and self-enhancement values broadens the discourse on the antecedents influencing domestic consumption behaviors. While previous studies have focused on factors such as consumers’ psychological characteristics [36,37], normative beliefs [17], and product-specific factors [87], the fact remains that consumer values can have a significant impact on consumption behavior [10,11,12,13,14]. Our literature review reveals a scarcity of studies that delve into how values influence domestic consumption behavior, with established studies focusing more on collectivist and individualist values. However, this categorization is relatively limited in its conceptualization [8] and fails to capture the content of more general human values. At the same time, much of the specific research tends to view collectivism or individualism as a universal characteristic of society as a whole [88,89,90] and cannot reflect individual consumer values. This paper applies Schwartz’s value theory, which is critical to this quest as it surveys citizens’ values from over 70 countries around the world to reflect a more generalized system of human values [47,63,64]. By analyzing values of self-transcendence, characterized by benevolence and universality, and self-enhancement, associated with the pursuit of power and achievement, this research not only extends the investigation into the antecedents of domestic-product consumption but also deepens the understanding of how consumer values, from a broader human value system, influence domestic-product consumption.

This study introduces the Norm-Activation Model (NAM) as a novel psychological explanatory mechanism, extending beyond the traditional focus on singular mediating variables to elucidate the dynamic process of norm activation. Previous research has identified factors such as attitudes [91], beliefs [92], and social norms [93] as mediating mechanisms in the impact of consumer values on consumption behavior. Additionally, numerous studies have highlighted the influence of values on consumption without delving into their psychological underpinnings [7,9,94]. This paper introduces personal norms to explain the intrinsic mechanism by which values influence behavior, and personal norms can be understood as internalized social norms that can have a more direct impact on behavior [22,23,24,25]. More importantly, rather than treating “personal norms” as a single mediating variable, this paper examines how values influence the dynamics of norm activation. Our model outlines how values influence consumption intentions through a sequence of mediators—Awareness of Consequence, Ascription of Responsibility, and Personal Norms. At the same time, we found that past research on how the NAM predicts pro-social or consumer behavior treats the NAM as a direct influence or as a variable alongside other factors [69,95,96,97], with few studies exploring the NAM’s role as a mediating mechanism. This innovative approach significantly expands the application of norm-activation theory within consumer behavior research.

The study validates the applicability of the mediation model of normative activation theory in a domestic consumption scenario. There are two dominant models between the variables of normative activation theory, and the divergence lies in the discussion about the relationship between two important variables, Ascription of Responsibility and Awareness of Consequence [70]. The mediation model posits that Awareness of Consequence triggers Ascription of Responsibility, thereby activating personal norms that subsequently impact individual behavior [70,84]. Conversely, the moderating model suggests that Awareness of Consequence and Ascription of Responsibility moderate the influence of personal norms on behavior [22,70]. Through structural-equation modeling, this study validates the NAM’s mediation model in the realm of domestic consumption and reinforces the mediation perspective by delineating the causal relations among NAM variables via the Dematel causality test. This aligns with De Groot’s findings across various pro-social behavioral settings [70] and echoes the NAM’s validation in scenarios of pro-environmental consumer behaviors, such as green purchasing [68] and food-waste reduction [97]. Consequently, the results not only bolster the mediation model’s applicability in consumption contexts but also underscore the predictive efficacy of normative activation theory in guiding consumers’ responsible-consumption practices.

### 5.2. Practical Contributions

These insights provide critical guidance for businesses aiming to engage customer segments with distinct value orientations, crafting marketing strategies that resonate deeply with these values. Brands that underscore self-transcendence values, which prioritize universalism and benevolence, are likely to positively influence consumers’ intentions toward domestic consumption. Such values should be highlighted by national brands or products targeting local customers. In the past, national brands might have leaned on promoting collectivism and patriotism in their advertising efforts. However, evidence suggests this approach might alienate certain consumer groups [98]. Our findings suggest that advertising campaigns emphasizing ”equality, mutual assistance, and societal concern” could potentially foster domestic purchasing behavior effectively. This approach presents a more neutral publicity route than patriotic advertising while potentially yielding similar benefits. Conversely, marketing slogans akin to Nike’s ”Just do it”, which underscores self-enhancement, may not resonate as effectively with local consumers who hold community-oriented values. In practical terms, national brands could adopt cause-related marketing strategies [99,100] and demonstrate active social responsibility [98], thereby showcasing their charitable and socially concerned ethos to attract the targeted local customer base.

Moreover, while prior research has highlighted the role of social norms in shaping intentions to consume domestic products [20,21], our study unveils the significant predictive power of personal norms on these intentions. The successful incorporation of norm-activation theory in our analysis offers fresh insights into strategies for promoting domestic consumption. For marketers, these findings underline the necessity of crafting strategies that influence the development of consumers’ personal norms. Such strategies could focus on highlighting the outcomes and importance of consumer behaviors or nurturing a sense of responsibility among consumers. For instance, associating the purchase of national products with the preservation of traditional culture and strengthening national brands can evoke a sense of duty and awareness of consequences in consumers. Through these approaches, businesses can effectively shape the formation of consumers’ personal norms, thus strategically directing patterns of domestic consumption.

### 5.3. Limitations and Future Research

This study, while insightful, is not without limitations, and these present avenues for future research.

Firstly, our research primarily examines the impact of values on domestic-product consumption among Chinese consumers. However, consumer behavior varies by country, culture, availability, knowledge, and experience [77]. Prior studies have shown that intentions to consume domestic products can significantly differ across cultural contexts [101] and the nature and intensity of values also vary by culture and region [46,102]. Therefore, to validate and generalize our findings, further research across diverse cultural and regional settings is necessary.

Secondly, the study acknowledges methodological limitations, particularly our reliance on survey data, which may introduce social desirability bias [103,104], potentially leading consumers to overestimate their willingness for local consumption. Social desirability bias may also explain some of the gap between intentions and behaviors. Meanwhile, values can be manipulated [105]. Practice allows for behavioral interventions on consumer values and norm-activation processes. Therefore, future research could benefit from experimental manipulations and field experiments to bolster the findings.

Moreover, in examining the causal relationships within the Norm-Activation Model (NAM), our study aligns with previous findings [70,84] regarding the strong link between awareness of consequences, ascription of responsibility, and personal norms [106,107]. Yet, recent studies suggest that when NAM variables are expanded, the path coefficients among original variables decrease [77]. Integrating the Theory of Planned Behavior (TPB) with the NAM has yielded better predictions of pro-environmental behaviors in some studies [108,109,110], suggesting a path for future research to explore expanded models of the NAM in predicting responsible and domestic consumption behavior.

Finally, this study does not account for potential contextual factors that might moderate the relationship between personal values and domestic-product-consumption intentions. Previous research highlights the influence of demographic factors on consumer values and their predictive power for responsible consumption behavior [42], noting differences in value orientations across age and gender [7]. Future studies should consider the impact of education, occupation, product attributes [87], and country-of-origin perceptions [37,111] on consumption choices, as these could moderate the values–consumption relationship and present fruitful areas for further investigation.

## 6. Conclusions

Firstly, this study provides a comprehensive analysis of how different personal value orientations impact the intention to consume domestic products. Beyond the collectivist and individualist values confirmed by prior research [8], our findings reveal that self-transcendence values, which prioritize universalism and benevolence, exert a positive influence on these intentions. Conversely, self-enhancement values, centered around power and achievement, are shown to negatively affect them. This broadens the understanding and scope of values influencing consumer behavior. Moreover, our results align with previous studies that identified contrasting predictive effects of self-transcendence and self-enhancement values on individual behaviors [55,56,57], thereby extending the research into the impact of values on pro-social behaviors to include domestic-product consumption.

Secondly, our study sheds light on the mechanisms underlying these effects through the application of the Norm-Activation Model (NAM). We found that self-transcendence values amplify the awareness of consequences associated with not consuming domestic products. This heightened awareness in turn strengthens the ascription of responsibility for potential adverse outcomes, leading to a more robust internalization of personal norms. Such a process increases the propensity toward consuming domestic products. On the flip side, self-enhancement values reduce the intention to consume domestic products by a similar sequence of awareness of consequences, ascription of responsibility, and personal norms. Our research not only supports previous findings regarding the influence of personal values in shaping norms in new contexts [26,27,28,29] but also advances the discourse beyond the conventional view of norms as a singular mediating variable. Contrary to prior studies, our work delineates the dynamic process through which values impact personal norms, offering a more nuanced understanding of their interplay.

Thirdly, our study rigorously validates the NAM’s mediation model, revealing how personal values guide the purchase of domestic products. The direct causal relationship of NAM’s variables was sorted out through the Dematel causality test. Our findings suggest that personal values shape individual norms and directly influence consumption intentions. It is worth noting that awareness of consequence and ascription of responsibility play mediating roles that activate personal norms. This is consistent with the results of previous studies on the relationship between the variables of norm-activation theory [70,84], thus demonstrating the applicability of the mediation model of norm-activation theory in the domestic-goods consumption scenario.

Furthermore, this study offers significant insights for managers and marketing practitioners, suggesting that emphasizing values such as equality, community care, and compassion in marketing efforts can effectively attract local consumers. It advocates for local firms to engage in cause-related marketing and demonstrate a strong commitment to social responsibility. The study also emphasizes the importance of influencing the process of activation of consumer norms. It is important to develop consumers’ awareness of the consequences of making domestic purchases and their sense of responsibility, thus leading them to adopt more informed and value-oriented consumer behavior.

## Figures and Tables

**Table 1:** CSS2021 variable-measurement question items.

Variable		Measurement	
		No.	Item
Independent variable	Self-enhancement	D7a-3	Which of the following do you think best describes a person’s value? -More powerful or wealthy than others.
	Self-transcendence	D7a-others	Which of the following do you think best describes a person’s value? Other items ^1^.
Dependent variable	Domestic-product-consumption intention	D9b-5	I favor national brands.

^1^ All items except “more powerful or wealthy than others” are included.

**Table 2:** Differences in values in domestic-product-consumption intention.

Variable	Values	N	Mean	SD	*t*	*p*
Domestic-product-consumption intention	Self-transcendence	21	2.476	0.750	−3.033	0.002
	Self-enhancement	1659	2.973	0.746		

SD = standard deviation.

**Table 3:** Demographic profile of the respondents.

Demographic	Group	Frequency	Percentage
Gender	Male	130	41.10%
	Female	186	58.90%
Age	0–18	2	0.62%
	19–25	86	27.22%
	26–30	71	22.47%
	31–40	97	30.70%
	41–50	36	11.39%
	51–60	23	7.28%
	Above 60	1	0.32%
Education	Below Middle School	9	2.85%
	High School	14	4.43%
	Bachelor’s Degree	213	67.41%
	Master’s Degree	68	21.52%
	Doctoral Degree	12	3.80%
Career	Student	35	11.08%
	Nationalized Business	93	29.43%
	Office-bearer	11	3.48%
	Private Business	160	50.63%
	Foreign Enterprise	14	4.43%

**Table 4:** Basic statistical information and sources of scale test items.

Constructs	Items	Mean	SD	FL	Sources
Self-enhancement	Wealth is important to him. He wanted to have a lot of money and expensive things.	3.28	1.622	0.690	The Portrait Values Questionnaire (PVQ) [49]
	Having authority and responsibility versus giving orders is essential to him. He wants people to do what he says.	2.75	1.530	0.873	
	He always wants to be the decision-maker. He likes to be the leader.	2.70	1.466	0.857	
	Showing what he can do is essential to him. He wants to be admired.	3.60	1.623	0.782	
	Success is essential to him. He wants people to recognize him.	2.92	1.476	0.834	
	He thinks it is essential to be ambitious. He wants to prove himself.	2.50	1.404	0.712	
	Motivation is essential to him. He strives to be better than others.	3.32	1.548	0.778	
Self-transcendence	He believes it is essential that all people are treated equally. He believes that people deserve equal opportunities in life.	2.31	1.284	0.799	
	Listening to different opinions is important to him. Even if he disagrees with others, he tries to understand their views.	2.38	1.193	0.764	
	He firmly believes that people should want to protect nature. Protecting the ecosystem is essential to him.	2.34	1.228	0.804	
	He believes that the world should live in harmony. It is essential to him to advocate for peace among the peoples of the world.	2.32	1.254	0.840	
	He longs for everyone to be treated fairly, even those he does not know. Protecting the vulnerable is essential to him.	2.41	1.201	0.821	
	Adapting to nature’s environment is essential to him. He believes that man should not change nature.	2.57	1.292	0.706	
Awareness of consequence	Buying foreign brands instead of national brands will inhibit national brands from maturing and hinder the development and growth of national industries.	3.54	1.554	0.838	[70,83,84,85]
	Purchasing foreign brands instead of national brands will lead to vicious competition among domestic enterprises, deteriorate the economic environment, and hinder sustainable economic development.	3.55	1.711	0.862	
	Buying foreign brands instead of national brands creates import dependency and increases the monopoly of foreign products.	3.21	1.590	0.838	
Ascription of responsibility	If the purchase of foreign brands instead of national brands inhibits national brands from reaching maturity and hinders the development and growth of national industries, I am responsible.	3.57	1.648	0.878	
	If the purchase of foreign brands instead of national brands leads to vicious competition among domestic enterprises, deterioration of the economic environment, and impeded sustainable economic development, I am responsible.	3.54	1.775	0.875	
	If the purchase of foreign brands instead of national brands leads to the emergence of the “foreign goods illusion” and import dependency and aggravates the monopoly of the market by foreign products, I will be held responsible.	3.47	1.717	0.875	
Personal norms	Buying foreign brands instead of national brands is against my moral principles.	3.87	2.012	0.860	
	I would feel guilty buying foreign brands instead of national brands.	3.74	1.986	0.904	
	I think I should buy national brands rather than foreign brands.	3.48	1.951	0.900	
	I think it is essential to buy national brands rather than foreign brands.	3.49	1.902	0.889	
	Due to my values and principles, I feel obliged to buy national brands rather than foreign brands.	3.46	1.955	0.885	
Domestic-product-consumption intention	I always choose national brands over foreign brands when buying home appliances.	3.43	1.829	0.885	[82]
	I always choose national brands over foreign brands when buying sports shoes and clothing.	3.56	1.939	0.877	
	I always choose national brands over foreign brands when I buy daily chemical products.	3.49	1.905	0.877	

**Table 5:** Scale reliability analysis.

Variables	Cronbach’s Alpha	Item Count
self-enhancement	0.919	7
self-transcendence	0.907	6
awareness of consequence	0.882	3
ascription of responsibility	0.917	3
personal norms	0.949	5
behavior	0.911	3
	0.890	27

**Table 6:** Model fit test.

Items	Results
CMIN/DF	2.021
RMSEA	0.057
TLI	0.952
CFI	0.957
IFI	0.952

**Table 7:** Convergent validity.

Factor	AVE	CR	Amount
Factor1	0.628	0.921	7
Factor2	0.624	0.909	6
Factor3	0.716	0.883	3
Factor4	0.789	0.918	3
Factor5	0.788	0.949	5
Factor6	0.775	0.912	3
Total			27
N			316

**Table 8:** Discriminant validity.

	Factor1	Factor2	Factor3	Factor4	Factor5	Factor6
Factor1	-					
Factor2	−0.490	-				
Factor3	−0.389	0.582	-			
Factor4	−0.362	0.617	0.701	-		
Factor5	−0.505	0.701	0.848	0.754	-	
Factor6	−0.507	0.760	0.738	0.646	0.928	-

**Table 9:** Descriptive statistics and correlation analysis.

Variables	Mean	Standard Deviation	SE	ST	AC	AR	PN	CB
SE	3.009	1.252	1.000					
ST	2.389	1.027	−0.454 **	1.000				
AC	3.435	1.457	−0.354 **	0.520 **	1.000			
AR	3.525	1.587	−0.337 **	0.564 **	0.630 **	1.000		
PN	3.610	1.786	−0.477 **	0.651 **	0.776 **	0.703 **	1.000	
DCPI	3.495	1.743	−0.468 **	0.691 **	0.662 **	0.591 **	0.863 **	1.000

** *p* < 0.01; SE = self-enhancement; ST = self-transcendence; PN = personal norms; DCPI = domestic-product-consumption intention; AC = awareness of consequence; AR = ascription of responsibility.

**Table 10:** Results of the path tests.

Model Paths	Estimate	S.E.	C.R.	*p*	Decision
SE → AC	−0.156	0.059	−2.908	0.004	-
ST → AC	0.559	0.094	8.621	***	-
AC → AR	0.704	0.063	12.176	***	-
SE → PN	−0.124	0.048	−3.509	***	Support H2a
ST → PN	0.212	0.082	4.618	***	Support H2a
AR → PN	0.194	0.062	3.601	***	-
AC → PN	0.572	0.085	8.295	***	-
ST → DCPI	0.233	0.076	5.187	***	Support H1b
PN → DCPI	0.760	0.051	14.083	***	-
SE → DCPI	−0.029	0.042	−0.869	0.385	-

*** *p* < 0.001; SE = self-enhancement; ST = self-transcendence; PN = personal norms; DCPI = domestic-product consumption intention; AC = awareness of consequence; AR = ascription of responsibility.

**Table 11:** Mediation analysis with self-enhancement values as the independent variable.

Mediation Paths	Effect	SE	LLCI	ULCI	Decision
Total effect					
SE → DCPI	−0.6322	0.0695	−0.7690	−0.4955	Support H1a
Indirect effect					
SE → PN → DCPI	−0.2230	0.0434	−0.3138	−0.1419	Support H3a
SE → AC → PN → DCPI	−0.2106	0.0376	−0.2890	−0.1378	Support H4a
SE → AC → AR → PN → DCPI	−0.0727	0.0197	−0.1162	−0.0399	Support H5a

SE = self-enhancement; PN = personal norms; DCPI = domestic-product consumption intention; AC = awareness of consequence; AR = ascription of responsibility; LLCI = bootstrapping lower-level confidential interval; ULCI = bootstrapping upper-level confidential interval.

**Table 12:** Mediation analysis with self-transcendence values as the independent variable.

Mediation Paths	Effect	SE	LLCI	ULCI	Decision
Direct effect					
ST → DCPI	1.1578	0 0682	1.0236	1.2921	Support H1b
Indirect effect					
ST → PN → DCPI	0.3461	0.0434	0.0533	0.4536	Support H3b
ST → AC → PN → DCPI	0.3222	0.0484	0.2270	0.4164	Support H4b
ST → AC → AR → PN → DCPI	0.0706	0.0244	0.0334	0.1278	Support H5b

ST = self-transcendence; PN = personal norms; DCPI = domestic-product consumption intention; AC = awareness of consequence; AR = ascription of responsibility; LLCI = bootstrapping lower-level confidential interval; ULCI = bootstrapping upper-level confidential interval.

**Table 13:** Dematel causality test results.

	Degree of Influence	Degree of Being Influenced	Centrality	Degree of Cause	Weights
SE	0.449	0.000	0.449	0.449	0.054
ST	1.499	0.000	1.499	1.499	0.179
AC	1.401	0.560	1.961	0.840	0.234
AR	0.243	0.861	1.103	−0.618	0.132
PN	0.596	1.246	1.841	−0.650	0.220
DCPI	0.000	1.520	1.520	−1.520	0.182

SE = self-enhancement; ST = self-transcendence; PN = personal norms; DCPI = domestic-product consumption intention; AC = awareness of consequence; AR = ascription of responsibility.

## Data Availability

The data of Study 1 contained within Supplementary Materials.

## Supplementary Materials

The following supporting information can be downloaded at http://css.cssn.cn/.
